# The secretome of periodontal ligament stem cells from MS patients protects against EAE

**DOI:** 10.1038/srep38743

**Published:** 2016-12-07

**Authors:** Thangavelu Soundara Rajan, Sabrina Giacoppo, Francesca Diomede, Patrizia Ballerini, Michele Paolantonio, Marco Marchisio, Adriano Piattelli, Placido Bramanti, Emanuela Mazzon, Oriana Trubiani

**Affiliations:** 1 IRCCS Centro Neurolesi “Bonino-Pulejo”, Via Provinciale Palermo, contrada Casazza, 98124, Messina, Italy; 2 Stem Cells and Regenerative Medicine Laboratory, Department of Medical, Oral and Biotechnological Sciences, University “G. d’Annunzio”, Chieti-Pescara, via dei Vestini, 31, 66100, Chieti, Italy; 3 Department of Psychological, Health and Territorial Sciences, University “G. d’Annunzio” Chieti-Pescara, via dei Vestini, 31, 66100, Chieti, Italy; 4 Department of Medicine and Aging Science, University “G. d’Annunzio” Chieti-Pescara, via dei Vestini, 31, 66100, Chieti, Italy

## Abstract

Manipulation of stem cells or stem cells-derived secretome has emerged as a novel alternative therapeutic option for multiple sclerosis (MS). Here we show that human periodontal ligament stem cells (hPDLSCs)-derived conditioned medium (hPDLSCs-CM) and purified exosomes/microvesicles (hPDLSCs-EMVs) obtained from Relapsing Remitting (RR)-MS patients and healthy donors block experimental autoimmune encephalomyelitis (EAE), a mouse model of MS, by inducing anti-inflammatory and immunosuppressive effects in spinal cord and spleen, and reverse disease progression by restoring tissue integrity via remyelination in the spinal cord. We show that hPDLSCs-CM and hPDLSCs-EMVs reduce pro-inflammatory cytokines IL-17, IFN-γ, IL-1β, IL-6, TNF-α, and induce anti-inflammatory IL-10. In addition, apoptosis related STAT1, p53, Caspase 3, and Bax expressions were attenuated. Our findings unravel the immunosuppressive effects of hPDLSCs-CM and hPDLSCs-EMVs in EAE mice, and suggest simple alternative autologous source for patient-customized cell-free targeting treatment in MS patients.

Multiple sclerosis (MS) is a crippling chronic autoimmune inflammatory disease characterized by the infiltration of immune cells to the central nervous system (CNS), demyelination and axonal loss, which produce the development of neurological symptoms^1^. Reports say that MS affects more than 2.5 million people among the general population. Based on clinical characteristics, the clinical course of MS has been distinguished as 4 subtypes: relapsing-remitting, primary progressive, secondary progressive and progressive relapsing, and each of these courses might be mild, moderate or severe. Several immune-modulating drugs are available for relapsing-remitting MS; however, until now, there is no treatment approved by the U.S Food and Drug Administration (FDA) for progressive forms^2^.

A growing body of evidence from animal studies demonstrates the administration of mesenchymal stem cells (MSCs) as a potential alternative treatment for MS^3^,^4^,^5^,^6^. MSCs are a heterogeneous subset of pluripotent non hematopoietic stromal cells that can be isolated from many different adult tissues such as bone marrow and adipose, possess the potential to differentiate into various cell lineages^7^, and are capable of translocating into damaged areas to provide immunomodulatory effects^8^,^9^,^10^. Recently, it has been proposed that MSCs exert their therapeutic effects mainly through the paracrine signaling of exosomes/microvesicles (EMVs). These are small membrane vesicles released by a variety of cell types including MSCs^11^, containing functional cytokines and other proteins, lipids and nucleic acids, such as mRNA and microRNA. The soluble bioactive molecules present in the EMVs directly activate the target cells, suppress pro-inflammatory responses, modulate the immune system^12^, inhibit apoptosis and fibrosis, and stimulate tissue-intrinsic progenitor cells differentiation^13^. Indeed, few studies have reported the therapeutic nature of isolated EMVs or whole cell-conditioned medium of MSCs in both *in vitro* and *in vivo* models, such as limb ischemia, breast cancer and multiple sclerosis^14^,^15^,^16^,^17^. Administration of MSCs secreted products may provide a novel cell-free regenerative therapeutical approach in various diseases^18^,^19^ since clinical implementation of the MSCs constituents may circumvent some of the limiting factors related to stem cell based therapies, which include immune incompetency, carcinogenicity, requirement for *ex vivo* cell expansion, and costs^20^.

Given the difficulty to harvest MSCs and their quantity from bone marrow and adipose tissues^21^,^22^, successive attempts have been made to harvest MSCs from oral derived tissues^23^,^24^. We have isolated pluripotent stem cells from adult human periodontal ligament (hPDLSCs), which is a soft connective tissue located between the tooth root and the alveolar socket, with unique characteristics. We reported that these cells show high self-renewal capability and multipotency; indeed, they differentiate into osteogenic, adipogenic, and condrogenic lineages^25^,^26^,^27^,^28^. Moreover, it is widely accepted now that, in vertebrates, oral stem cells originate from neural crest^15^,^29^.

In this view, we recently reported that hPDLSCs express proteins that are not present in bone marrow (BM)-MSCs including CLPP, NQO1, SCOT1, a new isoform of TBB5 and DDAH1. These proteins are involved in cell cycle regulation and stress response, homing, detoxification, neurogenesis and neuronal function homeostasis^30^. Indeed, the regenerative capacity of transplanted hPDLSCs has been assayed on *in vivo* models of periodontal tissue degeneration^31^,^32^ and recently we demonstrated the efficiency of hPDLSCs in EAE mice model^6^. In the present study, we evaluated for the first time the regenerative and immunomodulatory properties of hPDLSCs-conditioned whole culture medium (hPDLSCs-CM) and purified EMVs (hPDLSCs-EMVs) obtained from RR-MS patients in EAE mice and compared them with hPDLSCs-CM and hPDLSCs-EMVs obtained from healthy donors in order to evaluate the potential autologous therapeutic efficacy. To this end, we reported the characterization of the RR-MS-hPDLSCs in terms of expression of stemness markers, morphological features, proliferation rate and capability to differentiate into osteogenic and adipogenic lineages in comparison with hPDLSCs derived from healthy donors. Furthermore, we studied, *in vivo,* the clinical score and body weight, myelin regeneration and dendritic parameters, modulation of the anti-inflammatory immune responses, and regulation of apoptosis in spinal cord and/or spleen of EAE mice model systemically administered with RR-MS patients or donors hPDLSCs-CM and purified EMVs.

## Results

### Cytofluorimetric characterization of hPDLSCs and RR-MS-hPDLSCs

**Figure 1:**
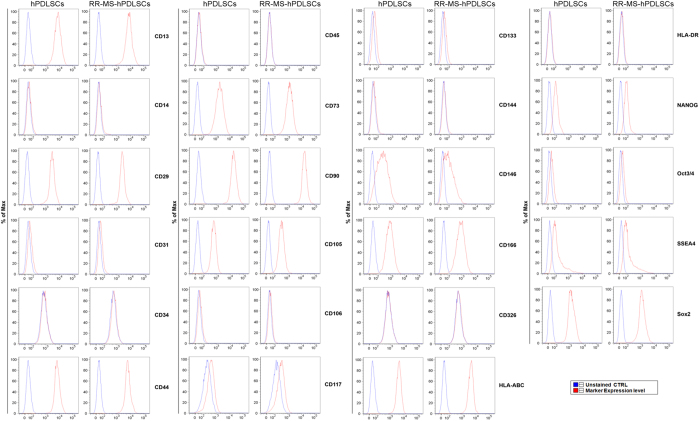
Flow cytometry of hPDLSCs and RR-MS-hPDLSCs phenotypes. Flow cytometry phenotype of surface related antigen of hPDLSCs and RR-MS-hPDLSCs at the 2^nd^ passage (CD13, CD14, CD29, CD31, CD34, CD44, CD45, CD73, CD90, CD105, CD106, CD117, CD133, CD144, CD146, CD166, CD326, HLA-ABC, HLA-DR) and intracellular stemness (SSEA4, Oct3/4, Sox2, NANOG) marker expression levels were detected. Red histograms show the distribution of each antigen expression, whereas Blue histograms represent the distribution of the respective background control. Data are representative of five separate experiments.

Figure 1 shows the positivity of the following surface markers inhPDLSCs and RR-MS-hPDLSCs: CD13, CD29, CD44, CD73, CD90, CD105, CD106, CD146, CD166, and HLA-ABC. Moreover, both hPDLSCs and RR-MS-hPDLSCs express pluripotency associated markers NANOG, Oct3/4, SSEA4, and SOX2.

### Morphological investigations of hPDLSCs and RR-MS-hPDLSCs

**Figure 2:**
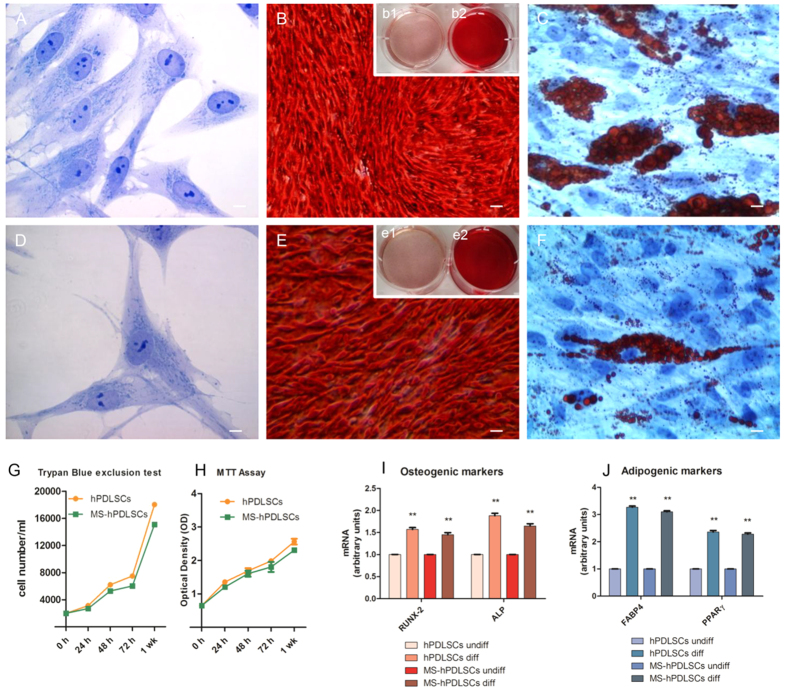
Toluidine blue staining of primary cultures of hPDLSCs observed by light microscopy. The cells display a fibroblast-like appearance with long cytoplasmatic processes, euchromatic nuclei with one or more nucleoli and rough endoplasmic reticulum profiles in both hPDLSCs [**A**] and RR-MS-hPDLSCs [**D**] samples. Original magnification: 40X. Cell proliferation and viability are assessed by Trypan blue exclusion test and MTT assay respectively [**G** and **H**]. The results are expressed as mean ± SEM of five independent experiments, and five replicates for each experimental point. hPDLSCs, and RR-MS-hPDLSCs induced to osteogenic differentiation were stained with Alizarin Red S after 3 weeks of culture [**B** and **E**, respectively]. Insets display uninduced hPDLSCs from healthy donors [b1] and RR-MS patients [e1]; b2 and e2 show differentiated hPDLSCs and RR-MS-hPDLSCs, respectively. High levels of mineralization are evident in the above mentioned samples. Original magnification: 10×. The bar graph shows mRNA levels, determined by real-time PCR, of osteo-related genes, i.e., alkaline phosphatase (*ALP*) and Runt-related transcription factor-2 (*RUNX2*) at 7 days of culture [**I**]. Adipogenic differentiation has been evaluated by the appearance of oil-red O-positive lipid vacuoles. [**C** and **F**, magnification: 40X]. The adipo-related genes, i.e., fatty acid binding protein 4 (*FABP4*) and peroxisome proliferator-activated receptor γ (*PPARγ*), analyzed by real-time PCR are shown [**J**]. The related mesengenic differentiation gene were over expressed in similar manner in hPDLSCs, and RR-MS- hPDLSCs. **p < 0.05. Scale bars = 10 μm. For each experiment, a representative image has been shown.

Primary cultures of hPDLSCs and RR-MS-hPDLSCs at 2^nd^ passage were observed at light microscopy. Adherent cells to glass coverslips showed a spindle-shaped morphology, with elongated cytoplasmic processes, both hPDLSCs and RR-MS-hPDLSCs cultures did not show any morphological differences [Fig. 2A and D, respectively].

### Cell growth and viability of hPDLSCs and RR-MS-hPDLSCs

Analysis from trypan blue assay of hPDLSCs and RR-MS-hPDLSCs, at 2^nd^ passage, no showed differences in viable cell number during the following culture time: 24, 48, 72 h and 1 week [Fig. 2G]. At the same time points MTT assay was performed [Fig. 2H], to evaluate the cell proliferation. The results show that hPDLSCs and RR-MS-hPDLSCs maintained the same rate in terms of number of viable cells and their proliferation.

### Mesengenic differentiation ability of hPDLSCs and RR-MS-hPDLSCs

Confluent living cells were induced to osteogenic differentiation, and after 3 weeks the cells were stained with Alizarin Red S to visualize the calcium deposits. The hPDLSCs and RR-MS-hPDLSCs showed several areas with mineralization deposits related to calcium deposition [Fig. 2B and E, respectively]. The analysis of transcripts RUNX-2 and ALP confirmed that hPDLSCs and RR-MS-hPDLSCs possess the similar ability to undergo versus osteogenic commitment [Fig. 2I]. To evaluate the adipogenic differentiation of hPDLSCs and RR-MS-hPDLSCs cellular monolayer were stained with Oil Red O and observed at light microscopy. Adipogenic-induced cells showed the evident intracellular lipid in both cultures [Fig. 2C and F, respectively]. These data were supported by RT-PCR that demonstrated an up-regulation of FABP4 and PPARγ, molecules that influence the adipogenesis differentiation process [Fig. 2J].

### Analysis of EMVs from conditioned medium of hPDLSCs and RR-MS-hPDLSCs

**Figure 3:**
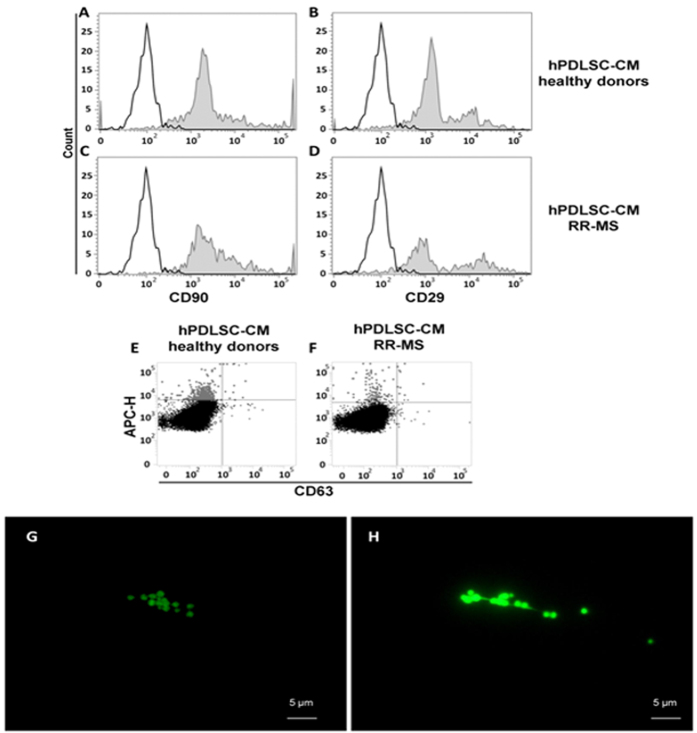
Characterization of EMVs detected in hPDLSCs and RR-MS-hPDLSCs conditioned medium. Filled histograms show the distribution of CD29 and CD90 antigen expression, whereas open histograms represent the distribution of the respective background control in hPDLSCs [**A** and **B**] and RR-MS-hPDLSCs [**C** and **D**]. The expression of CD63 was evaluated in combination with Mitotracker to exclude debris [**E** and **F**]. Data are representative of five separate experiments. The characterization of isolated EMVs stained with WGA derived from hPDLSCs and RR-MS-hPDLSCs is reported in section [**G** and **H**, respectively]. Different size in the parental EMVs isolated with the ExoQuick-TC method was observed. Scale bars: 5 μM.

Figure 3 shows the expression of surface mesenchymal related molecules (CD29, CD90) in whole conditioned medium (CM) from hPDLSCs [Fig. 3A and B, respectively] and RR-MS-hPDLSCs [Fig. 3C and D, respectively]. The histograms display no difference between the samples examined.

Moreover, exosome subpopulations in the CM was evidenced using the CD63 exosome marker in hPDLSCs [Fig. 3E] and in RR-MS-hPDLSCs [Fig. 3F] EMVs. Fluorescent lipid probes (Alexa Fluor® 488 conjugate Wheat germ agglutinin) was used to track the enriched EMVs obtained through ExoQuick-TC assay from hPDLSCs and RR-MS-hPDLSCs displayed a subcellular spherical morphological structure [Fig. 3G and H, respectively]. The present data provided clear evidence that the ExoQuick-TC methods permits to obtain high level of EMVs concentration.

### Intravenous administration of RR-MS patients and healthy donors hPDLSCs-CM and purified EMVs reduce clinical disease scores, and augment spine density and remyelination in the spinal cord of EAE mice

**Figure 4:**
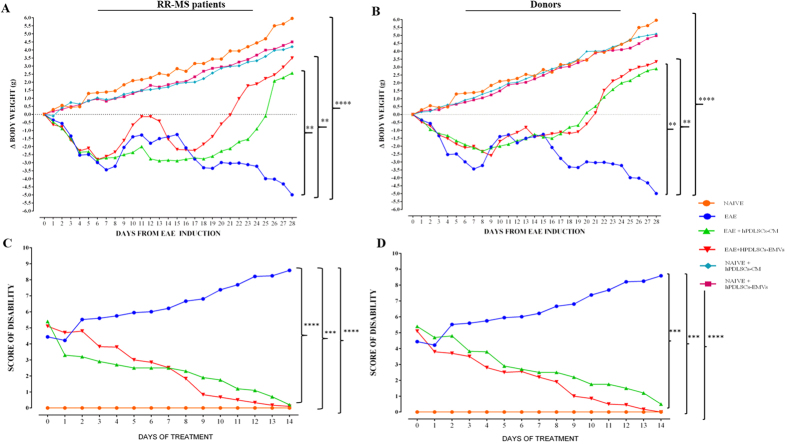
Body weight and clinical score. Mice were immunized with MOG _35–55_ and monitored 28 days for body weight gain/loss and clinical disease score. (**A** and **B**) The variation of body weight has been expressed compared to day of EAE induction (day 0) for each experimental group. Naive mice showed a normal increase in body weight. On the contrary, a significant body weight loss was observed in EAE mice, whereas a relevant body weight gain was found in EAE mice treated with RR-MS patients human periodontal ligament stem cells (hPDLSCs)-derived conditioned medium (hPDLSCs-CM) or purified exosomes/microvesicles (hPDLSCs-EMVs) [**A**; ****p < 0.0001 vs EAE; **p = 0.0029 vs EAE + hPDLSCs-CM; **p = 0.0030 vs EAE + hPDLSCs-EMVs] and in EAE mice treated with donors hPDLSCs-CM or hPDLSCs-EMVs [**B**; ****p < 0.0001 vs EAE; **p = 0.0025 vs EAE + hPDLSCs-CM; **p = 0.0026 vs EAE + hPDLSCs-EMVs]. (**C** and **D**) Naive mice did not display motor deficit. EAE mice displayed a grading of disease, while significant reduction in the clinical scores was observed in EAE mice treated with RR-MS patients hPDLSCs-CM or hPDLSCs-EMVs [**C**; ****p < 0.0001 vs EAE; ***p < 0.0009 vs EAE; ****p < 0.0001 vs naive] and in EAE mice treated with donors hPDLSCs-CM or hPDLSCs-EMVs [**D**; ***p < 0.0008 vs EAE; ***p < 0.0002 vs EAE; ****p < 0.0001 vs naive].

**Figure 5:**
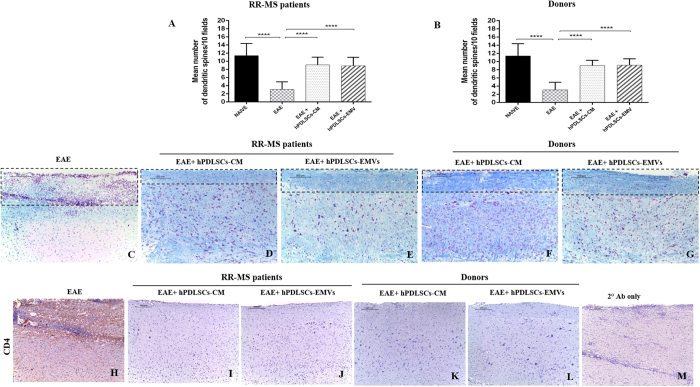
Golgi staining, LFB staining and CD4 expression in the spinal cord. Dendritic spines were reduced in EAE mice, whereas EAE mice treated with hPDLSCs-CM or hPDLSCs-EMVs obtained from RR-MS patients [**A]** and donors [**B]** showed significant augmentation in spine density. In addition, EAE mice without treatment exhibited remarkably reduced myelin in the spinal cord [**C**; area showed in dotted square], whereas treatment with hPDLSCs-CM or hPDLSCs-EMVs obtained from RR-MS patients [**D** and **E**, respectively] and donors [**F** and **G**, respectively] reduced demyelination and axonal loss in EAE mice with an intense LFB positive staining. CD4 [**H**] expression revealed marked positive staining in EAE mice, while EAE mice administered with hPDLSCs-CM or hPDLSCs-EMVs derived from MS patients [**I** and **J**, respectively] and donors [**K** and **L**, respectively] showed negative staining. [**M]** only secondary antibody.

Body weight and clinical score are directly associated with the progression of EAE. In our study, naive mice showed a normal increase in body weight, while a significant body weight loss was observed in MOG_35–55_ injected mice, which indicates the severe EAE development. On the contrary, relevant body weight gain was found in EAE mice treated with MS patients hPDLSCs-CM and hPDLSCs-EMVs [Fig. 4A], and in EAE mice treated with healthy donors hPDLSCs-CM or hPDLSCs-EMVs [Fig. 4B]. The body weight gain in EAE mice treated with MS patients and donors hPDLSCs-CM and hPDLSCs-EMVs was paralleled by the amelioration of clinical score. Naive mice did not display motor deficit. EAE mice displayed a grading of disease, while significant reduction in the clinical scores was observed in EAE mice treated with RR-MS patients hPDLSCs-CM or hPDLSCs-EMVs [Fig. 4C] and in EAE mice treated with donors hPDLSCs-CM or hPDLSCs-EMVs [Fig. 4D]. In addition, we quantified the number of dendritic spines in EAE mice and EAE mice treated with hPDLSCs-CM or hPDLSCs-EMVs obtained from MS patients and donors. Dendritic spines were reduced in EAE mice, whereas EAE mice treated with hPDLSCs-CM or hPDLSCs-EMVs obtained from MS patients [Fig. 5A] and donors [Fig. 5B] showed significant augmentation in spine density. Moreover, EAE mice without treatment exhibited remarkably reduced myelin in the spinal cord [Fig. 5C], whereas treatment with hPDLSCs-CM or hPDLSCs-EMVs obtained from MS patients [Fig. 5D and E, respectively] and donors [Fig. 5F and G, respectively] reduced demyelination and axonal loss in EAE mice with an intense LFB positive staining.

### Intravenous administration of RR-MS patients and healthy donors hPDLSCs-CM and purified EMVs induce anti-inflammatory and immunosuppressive effects in the spinal cord of EAE mice

**Figure 6:**
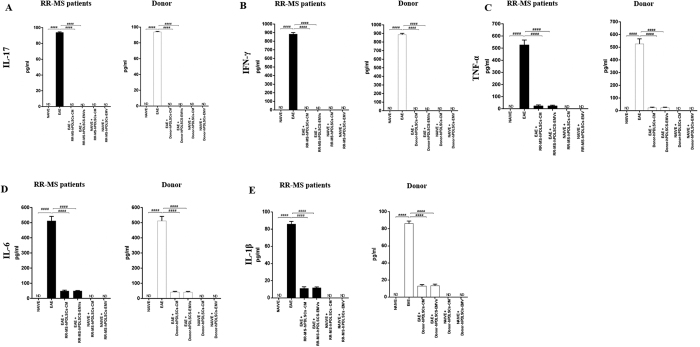
ELISA assay for inflammatory cytokines in the spinal cord. ELISA data demonstrated that proinflammatory cytokines IL-17 [**A**], IFN-γ [**B**], TNF-α [**C**], IL-6 [**D**] and IL-1β [**E**] were markedly increased in EAE mice, while administration of RR-MS patients and donors derived hPDLSCs-CM and hPDLSCs-EMVs significantly reduced their expression. The data are representative of three independent experiments. Error bars indicate mean ± SD. ^####^p < 0.0001.

**Figure 7:**
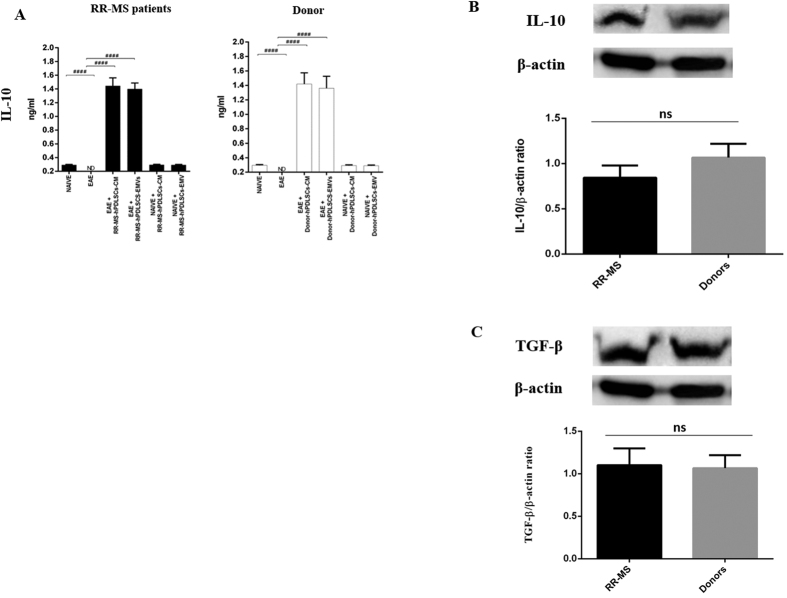
ELISA assay for IL-10 in the spinal cord and Western blot analysis for IL-10 and TGF-β in hPDLSCs-EMVs. Anti-inflammatory IL-10 was undetectable in EAE mice, while administration of hPDLSCs-CM or hPDLSCs-EMVs derived from RR-MS patients and donors siginificantly augmented IL-10 expression [**A**]. The data are representative of three independent experiments. Error bars indicate mean ± SD. ^####^p < 0.0001. Western blot analysis in the extract of hPDLSCs-EMVs isolated from healthy donors and RR-MS patients showed a marked level of IL-10 [**B**] and TGF-β [**C**]. IL-10 and TGF-β were quantified by densitometry after normalizing the bands to β-actin. No statistical significance in IL-10 and TGF-β expression was noticed between RR-MS patients and donors hPDLSCs-EMVs. A representative graph of three independent experiments is shown. ns-no statistical significance.

Then we assessed the anti-inflammatory and immunosuppressive effects of MS patients and healthy donors hPDLSCs-CM and hPDLSCs-EMVs in the spinal cord of EAE mice. Immunohistochemistry results showed prominent positive staining for pro-inflammatory CD4 [Fig. 5H] in EAE mice, while EAE mice administered with hPDLSCs-CM or hPDLSCs-EMVs derived from MS patients [Fig. 5I and J, respectively] and donors [Fig. 5K and L, respectively] showed negative staining. ELISA results showed upregulation of pro-inflammatory cytokines IL-17 [Fig. 6A], IFN-γ [Fig. 6B], TNF-α [Fig. 6C], IL-6 [Fig. 6D], and IL-1β [Fig. 6E] in EAE mice, while administration of hPDLSCs-CM and EMVs derived from MS patients and donors suppressed the activation of these cytokines in EAE mice. On the contrary, anti-inflammatory cytokine IL-10 was significantly elevated in MS patients and donors hPDLSCs-CM and EMVs treated EAE mice. IL-10 was not detectable in EAE mice [Fig. 7A]. ELISA results were further confirmed with immunohistochemistry experiments (See Supplementary Figure 1 for IL-17 and IFN-γ, 2 for IL-1β and IL-6, and 3 for TNF-α and IL-10.).

### RR-MS patients and healthy donors-derived EMVs contain IL-10 and TGF-β

In order to understand the mechanistic cues associated with the anti-inflammatory effects of CM and EMVs, we performed Western blot analysis in the extract of EMVs isolated from hPDLSCs of healthy donors and RR-MS patients. We found marked level of IL-10 [Fig. 7B] and TGF-β [Fig. 7C] present in EMVs. No statistical significance in IL-10 and TGF-β expression was noticed between RR-MS patients and donors EMVs. These results suggested that the anti-inflammatory effect of hPDLSCs-derived CM or EMVs might be due to the presence of immunomodulatory cytokines IL-10 and TGF-β.

### Intravenous administration of RR-MS patients and healthy donors hPDLSCs-CM and purified EMVs attenuate apoptosis in the spinal cord of EAE mice

**Figure 8:**
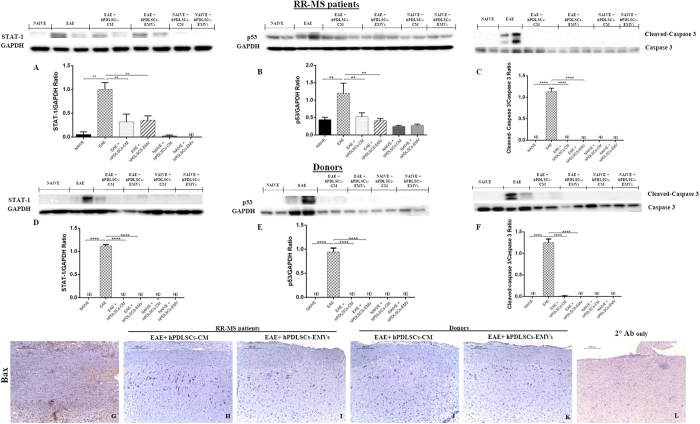
Western blot analysis for STAT1, p53, and cleaved caspase 3, and immunohistochemical evaluation for Bax in the spinal cord. Western blot analysis showed a significant expression of proapoptotic STAT1 [**A** and **D**] and p53 [**B** and **E**] in EAE mice. Conversely, levels of STAT1 and p53 were significantly reduced by administration of hPDLSCs-CM or hPDLSCs-EMVs derived from MS patients and donors. A **p < 0.0011 vs naive, **p < 0.0070 vs CM, **p < 0.0093 vs EMVs. D ****p < 0.0001. B **p < 0.0047 vs naive, **p < 0.0056 vs CM, **p < 0.0081 vs EMVs. E ****p < 0.0001. Moreover, EAE caused a significant increase in proapoptotic cleaved caspase 3 expression. On the contrary, treatment with hPDLSCs-CM or hPDLSCs-EMVs derived from MS patients [**C**] and donors [**F**] significantly prevented the EAE-induced cleaved caspase 3 expression. C ****p < 0.0001. F ****p < 0.0001. STAT1, p53 and cleaved caspase 3 were quantified by densitometry after normalizing the bands to GAPDH or caspase 3. Representative bands of three independent experiments are shown. Error bars indicate mean ± SD. ND: not detectable. Immunohistochemical evaluation revealed an increased Bax tissue localization in EAE mice [**G**]. On the contrary, negative staining of Bax was observed in mice that received hPDLSCs-CM or hPDLSCs-EMVs derived from MS patients [**H** and **I**, respectively] and donors [**J** and **K**, respectively]. Magnification 10X. L only secondary antibody.

Then we investigated whether anti-inflammatory and immunosuppressive effects promoted by hPDLSCs-CM or hPDLSCs-EMVs may induce anti-apoptotic effects. Western blot analysis showed a significant expression of STAT1 [Fig. 8A and D] and p53 [Fig. 8B and E] in EAE mice. Conversely, levels of STAT1 and p53 were significantly reduced by administration of hPDLSCs-CM or hPDLSCs-EMVs derived from MS patients and donors. Moreover, EAE caused a significant increase in cleaved caspase 3 expression. On the contrary, treatment with hPDLSCs-CM or hPDLSCs-EMVs derived from MS patients [Fig. 8C] and donors [Fig. 8F] significantly prevented the EAE-induced cleaved caspase 3 expression. GAPDH was used to assess the equal protein loading. In addition, immunohistochemical evaluation revealed an increased Bax tissue localization in EAE mice [Fig. 8G]. On the contrary, negative staining of Bax was observed in mice that received hPDLSCs-CM or hPDLSCs-EMVs derived from MS patients [Fig. 8H and I, respectively] and donors [Fig. 8J and K, respectively]. These results demonstrated the significant anti-apoptotic effects of hPDLSCs-CM or hPDLSCs-EMVs obtained from MS patients and healthy donors.

### Intravenous administration of RR-MS patients and healthy donors hPDLSCs-CM and purified EMVs induce anti-inflammatory and immunosuppressive effects in the spleen of EAE mice

**Figure 9:**
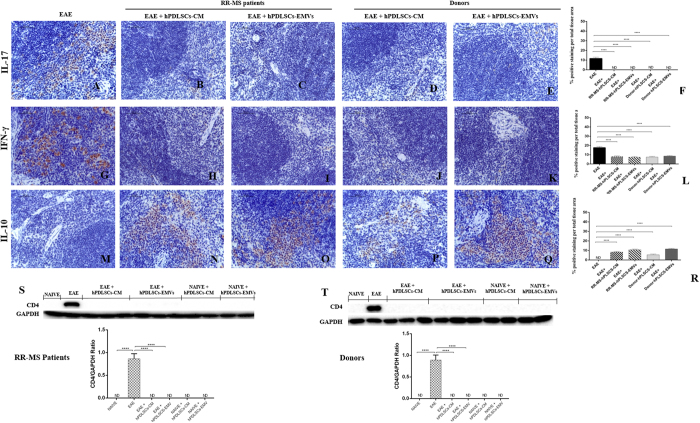
Modulation of inflammatory cytokines in the spleen. Immunohistochemistry analyses of pro-inflammatory IL-17 [**A**; densitometric analysis **F**] and IFN-γ [**G**; densitometric analysis **L**] revealed higher tissue levels of these markers in EAE mice, while significant reduction was noticed in EAE mice administered with hPDLSCs-CM or hPDLSCs-EMVs derived from RR-MS patients [**B**,**C** and **H**,**I**, respectively] and donors [**D**,**E** and **J**,**K**, respectively]. EAE sections did not stain for anti-inflammatory IL-10 antibody [**M;** densitometric analysis **R**], while significant positive IL-10 staining was noticed in the EAE group treated with hPDLSCs-CM or hPDLSCs-EMVs obtained from MS patients [**N** and **O**, respectively] and donors [**P** and **Q**, respectively]. ****p < 0.0001 vs EAE. Magnification 40X. Western blot analysis showed a significant expression of pro-inflammatory CD4 in the spleen of EAE mice [**S** and **T**, respectively]. Conversely, as in naive animals, CD4 was undetectable in the EAE group treated with hPDLSCs-CM or hPDLSCs-EMVs obtained from RR-MS patients and donors. CD4 was quantified by densitometry after normalizing the bands to GAPDH. Representative bands of three independent experiments are shown. Error bars indicate mean ± SD. ****p < 0.0001.

In our study, also we investigated whether hPDLSCs-CM or hPDLSCs-EMVs obtained from MS patients and healthy donors may induce anti-inflammatory responses in peripheral system as well. To this end, we evaluated pro-inflammatory markers IL-17, IFN-γ, CD4, and anti-inflammatory marker IL-10 in the spleen. Immunohistochemical analyses for IL-17 [Fig. 9A; densitometric analysis Fig. 9F] and IFN-γ [Fig. 9G; densitometric analysis Fig. 9L] expression revealed marked positive staining for these markers in EAE mice, while negative staining was noticed in EAE mice treated with hPDLSCs-CM or hPDLSCs-EMVs derived from MS patients [Fig. 9B,C and H,I, respectively] and donors [Fig. 9D,E and J,K, respectively]. On the other hand, EAE sections did not stain for anti-inflammatory IL-10 antibody [Fig. 9M; densitometric analysis Fig. 9R], while significant positive IL-10 staining was noticed in the EAE group treated with hPDLSCs-CM or hPDLSCs-EMVs obtained from MS patients [Fig. 9N and O, respectively] and donors [Fig. 9P and Q, respectively]. For secondary antibody control immunohistochemistry images of IL-17, IFN-γ and IL-10, see Supplementary Figure 4. Western blot analysis showed a significant expression of pro-inflammatory CD4 in the spleen of EAE mice. Conversely, as in naive animals, CD4 was undetectable in the EAE group treated with hPDLSCs-CM or hPDLSCs-EMVs obtained from MS patients [Fig. 9S] and donors [Fig. 9T]. These results demonstrated that hPDLSCs-CM and hPDLSCs-EMVs derived from MS patients and healthy donors could produce anti-inflammatory and immunosuppressive responses, not only in the spinal cord, but also in the spleen of EAE mice.

## Discussion

MS is a chronic autoimmune disease that affects the central nervous system, in which the immune system produces antibodies against the myelin sheath of nerve fibers, causing demyelination. The disease may be mild, moderate or severe, and most patients have the relapsing-remitting course. Although considered as a debilitating disease, there is still no FDA-approved treatment for progressive MS and drugs are available only to reduce the rate of occurrence and intensity of relapses, and to mitigate disease progression^2^. In recent years, MSCs have been demonstrated as a novel alternative therapeutic option for MS due to their ability to suppress inflammatory/autoimmune responses and to limit the appearance of neurological symptoms in animal models associated with MS^4^,^6^,^10^,^16^,^17^. Difficulties in isolating MSCs from bone marrow and adipose tissues have made clinicians eager to look for alternative tissue sources for stem cells such as periodontal ligament tissue^26^. Recently, we showed for the first time that hPDLSCs promotes polymorphonuclear neutrophils (PMN) survival and reduces bactericidal activity, and that cell-cell contact was not strictly necessary for these effects to take place, indicating paracrine immunoregulatory functions of hPDLSCs^33^. Indeed, they release cytokines and growth factors (i.e., IL-8, VEGF, and IL-6)^34^ that promote PMN recruitment and survival^35^,^36^. They also release IL-10, which limits the pro-inflammatory activities of PMN^37^ and improves wound repair during periodontitis^33^,^38^.

To our best knowledge, in the present study we showed for the first time that the primary cultures of stem cells from healthy human periodontal ligaments are similar to those from RR-MS-hPDLSCs patients for their high clonogenic potential and for the expression of embryonic and mesenchymal markers. Morphological analysis demonstrated that RR-MS-hPDLSCs exhibited fibroblast-like morphology after *in vitro* expansion similar to hPDLSCs from healthy donors. Moreover, RR-MS-hPDLSCs show a similar proliferative ability, alike healthy PDLSCs, as pointed out in MTT assay. Moreover, we have investigated through cytofluorimetric analysis the markers associated to EMVs released from primary culture of hPDLSCs and RR-MS-hPDLSCs. EMVs are cell-derived membrane vesicles, and represent an endogenous mechanism for intercellular communication. Since the discovery that EMVs are capable of functionally transferring biological information, the potential use of EMVs as drug delivery vehicles has gained considerable scientific interest^39^. Our results showed that the EMVs present in CM are positive for surface mesenchymal antigens CD 90 and CD 29 and exosome related protein CD63. The vesicles are a mixed population of exosomes and shedding vesicles^40^,^41^. Indeed, in our study the fluorescence microscopic analyses revealed that the isolated EMVs show spherical morphology associated to a different size in both healthy and disease specific cells.

Based on these *in vitro* results and on our recent evidences^33^,^34^ we were encouraged to evaluate the potential therapeutic effects of autologous RR-MS-hPDLSCs-derived CM and purified EMVs *in vivo* in experimental autoimmune encephalomyelitis mice model, considered as a relevant preclinical model of MS. The *in vivo* results obtained confirmed our expectation. We showed that hPDLSCs-CM and hPDLSCs-EMVs obtained from both RR-MS patients and healthy donors were effective to significantly reduce the MOG-induced activation of CD4^+^ Th1 and Th17 cells in spinal cord and in spleen, an important peripheral lymphoid tissue target, in EAE mice. These results were further corroborated by the reduction of Th1/Th17 pro-inflammatory cytokines IFN-γ, IL-17, IL-6 and TNF-α. Indeed, similar reduction in T cell proliferation and IFN-γ release were previously reported in *in vitro* stimulated T cells treated with human adipose MSCs derived exosomes^42^. In addition, we observed a significant upregulation of anti-inflammatory cytokine IL-10, which denotes the possible activation of Th2 cells. We assume that the presence of EMV surface marker CD90 in the CM derived from RR-MS patients and healthy donors hPDLSCs may induce the production of IL-10 since the positive regulatory role of CD90 in IL-10 production has been already reported^43^.

Moreover, secretion of IL-10 by human embryonic and adipose tissue MSCs-derived CM and EMVs has been reported in *in vitro* studies with T lymphocytes cell lines^44^,^45^. Our *in vivo* findings support these results, provide compelling evidence for similar immunomodulatory characteristics of CM and EMVs derived not only from hPDLSCs of healthy donors, but more interestingly also from those of RR-MS patients. Moreover, the immunoprotective role of IL-10 in myelination and dendritic spines in EAE mice has been previously reported^46^,^47^,^48^. We thus speculate that the regenerative aspects, such as increased remyelination and dendritic spines measures of hPDLSCs-CM and EMVs, might be related to IL-10 production.

In order to understand further putative mechanisms underlying the anti-inflammatory effects, we analyzed the extract of hPDLSCs-derived EMVs isolated from donors and RR-MS patients. Immunomodulatory cytokines present in EMVs of different MSCs has been reported in previous studies^45^,^49^ In accord with these results, we noticed the presence of IL-10 and TGF-β in EMVs. Consequently, we suppose that the presence of IL-10 and TGF-β in both RR-MS patients and healthy donors EMVs may have crucial role in repressing the activation of pro-inflammatory signaling cascade in EAE mice.

Moreover, the reported similar immunosuppressive effects stimulated by both RR-MS patients and healthy donors might suggest the pathology-independent functional niche of hPDLSCs. Indeed, such pathology-independent functional feature of stem cell niche has been reported in MSCs derived from BM of patients affected by amyotrophic lateral sclerosis, another progressive neurodegenerative disease^50^. Further experiments on hPDLSCs-derived secretome obtained from patients affected by neurodegenerative diseases including Alzheimer’s disease, Parkinson’s disease etc., may provide substantial cues to investigate this hypothesis. Besides, it is worth mentioning that naive mice injected with RR-MS patients or donors hPDLSCs-CM or EMVs did not show any immune responses, which indicates the selective immunomodulatory role of hPDLSCs-CM and EMVs in EAE inflammatory microenvironment. Future study to investigate the cytokines and other immunomodulatory proteins present in hPDLSCs-CM and EMVs shall render more mechanistic insights associated with the suppression of inflammation and autoimmune responses in MS.

In addition, we demonstrated that RR-MS patients and healthy donors hPDLSCs-CM and EMVs could potentially exert apoptosis regulatory functions in EAE mice spinal cord. Pro-inflammatory cytokines are able to induce p53, and are involved in the enhancement of p53-mediated apoptosis. In this study, we investigated STAT1- p53- cleaved caspase 3- Bax pathway mediated apoptosis in EAE mice. As expected, activation of pro-inflammatory cytokines positively recruited STAT1, p53, cleaved caspase 3 and Bax proteins in EAE mice spinal cord. On the contrary, EAE mice administered with RR-MS patients or donors hPDLSCs-CM and EMVs showed significant reduction in STAT1, p53, cleaved caspase 3 and Bax expressions. Anti-apoptotic effects of adult MSCs derived CM and EMVs in *in vitro* hypoxia and *in vivo* cardiac and kidney injury models have already been examined^51^,^52^,^53^,^54^,^55^,^56^; however, most of these studies described the apoptosis regulatory effects in the context of microRNAs (miRNAs) present in MSCs secretions. From our results, we confirmed similar anti-apoptotic effects exerted from CM and EMVs of RR-MS patients and donors hPDLSCs. Further study to explore the miRNA profile of hPDLSCs shall provide more insight into their anti-apoptotic effects.

In summary, CM and purifed EMVs from both RR-MS patients and donors hPDLSCs elicited immunosuppressive effects in EAE mice. We speculate that the beneficial effects produced by conditioned medium might be attributed to its EMVs fractions, adding further support in the context of personalized regenerative stem-cell free therapy for MS. Stem-cell free therapy approach has been emerged as potentially safer and cost-effective alternatives for a wide range of diseases^57^,^58^,^59^,^60^. Our preclinical data presented here suggest that secretome derived from hPDLSCs may represent a unique autologous therapeutic source in the clinical application of RR-MS patients.

## Methods

### hPDLSCs *ex vivo* culture from healthy and RR-MS patient’s ethics statement

The study was performed in accordance with the guidelines of the Helsinki Declaration (2013). The protocol and data collection were approved by Ethics Committee at the Medical School, “G. d’Annunzio” University, Chieti, Italy (No. 266/17.04.14). The informed consent was obtained from all subjects before samples collection. The Department of Medical, Oral and Biotechnological Sciences and the Laboratory of Stem Cells and Regenerative Medicine are certified according to the quality standard ISO 9001:2008 (certificate No. 32031/15/S).

### Isolation, Culture and immunophenotyping of hPDLSCs and RR-MS-hPDLSCs

Five human periodontal ligament biopsies were scraped from human premolar teeth of healthy and Relapsing Remitting-Multiple Sclerosis (RR-MS) patients. The tissue was obtained by scaling the roots using Gracey’scurettes^34^. The samples were washed five times with PBS (LiStarFish), and cultured using TheraPEAK MSCGM-CD BulletKit serum free, chemically defined (MSCGM-CD) medium for the growth of human Mesenchymal Stem Cells (Lonza, Basel, Switzerland). The medium was changed twice a week, and cells migrating from the explants tissue after reaching about 80% of confluence, were trypsinized (LiStar Fish), and after subcultured until passage 2^nd^ (P2). 5 × 10^5^ hPDLSCs expanded from healthy and RR-MS patient’s (RR-MS-hPDLSCs) at the 2^nd^ passage were incubated with 1 μg of the specific antibody, conjugated with fluorescein isothiocyanate (FITC), phycoerythrin (PE), allophycocyanin (APC), phycoerythrin-cyanine 5.5 (PE Cy5.5), or Alexa Fluor 488 for 30 min at 4 °C in the dark. Cells were stained using the following antibodies: anti-CD13, anti-CD29, anti-CD44, anti-CD45, anti-CD105, anti-CD166 (Ancell, MN, USA), anti-CD14, anti-CD133 (BergischGladbach, Germany),anti-CD63, anti-CD73, anti-CD90, anti-CD117, anti-CD146, anti-CD271, anti-Sox2, anti-HLA-DR, anti-SSEA4, anti-OCT3/4 (Becton Dickinson, BD, San Jose, CA, USA); anti-CD144 (Acris Antibodies, Herford, Germany), anti-CD34 (Beckman Coulter, Fullerton, CA, USA). After incubation, cells were acquired with a flow cytometer (FACS Calibur; BD). Data were analyzed by the FlowJo software v8.8.6 (TreeStar, Ashland, OR).

### Morphological analysis

Glass-adherent hPDLSCs and RR-MS-hPDLSCs at 2^nd^passage were observed with a Leica DMIL10 (Leica Microsystem, Milan, Italy) optical microscope, and images were captured using a Leica EC3 digital camera apparatus.

### Cell proliferation and viability assay

For trypan blue staining hPDLSCs and MS-hPDLSCs after 24, 48, 72 h and 1 week of culture were incubated with 0.5% of trypan blue solution for 10′ min at room temperature and subsequently analysed with Burker’s chamber.

For MTT assay, hPDLSCs and RR-MS-hPDLSCs were seeded at 2 × 10^3^ cells/well in triplicate using a 96-well flat-bottom plate and maintained in MSCGM-CD for 24, 48, 72 h and 1 week. After the incubation period, 15 μl/well of MTT were added to culture medium and cells were incubated for 3 h at 37 °C. The supernatants were read at 650 nm wavelength using a microplate reader (Synergy HT, BioTek Instruments, Vermont, USA). Moreover, the MTT and Trypan Blue assays were achieved in five independent experiments, one for each donor, and five replicates for each experimental point.

### Differentiation potential evaluation at morphological level

For osteogenic and adipogenic differentiation hPDLSCs and RR-MS-hPDLSCs at 2^nd^ passage were incubated in MSCGM-CD (Lonza, Walkersville, MD) medium added with osteogenic supplements and in adipogenesis induction/maintanance medium (Lonza) respectively. Osteogenic and adipogenic induction was confirmed by means colorimetric assay as previously described by Trubiani *et al*.^26^.

### RNA isolation and Real Time-PCR Analysis

To assess osteogenic and adipogenic differentiation ability of PDLSCs and RR-MS-hPDLSCs, total RNA was isolated using the Total RNA Purification Kit (NorgenBiotek Corp., Ontario, CA) according to the manufacturer’s instructions. The M-MLV Reverse Transcriptase reagents (Applied Biosystems) were used to generate cDNA. Real-Time PCR was carried out with the Mastercycler ep realplex real-time PCR system (Eppendorf, Hamburg, Germany). hPDLSCs expression of Runt-related transcription factor-2 (RUNX-2) and AlkalinPhospatase (ALP) was evaluated after 7 days in osteogenic differentiated culture, the expression of Fatty Acid Binding Protein 4 (FABP4) and Peroxisome Proliferator-Activated Receptor γ (PPARγ) were analysed after 28 days of adipogenic differentiation culture. Commercially available TaqMan Gene Expression Assays (RUNX-2 Hs00231692_m1; ALP Hs01029144_m1; FABP4 Hs01086177_m1; PPARγ Hs01115513_m1) and the TaqMan Universal PCR Master Mix (Applied Biosystems, Foster City, CA, USA) were used according to standard protocols. Beta-2 microglobulin (B2M Hs99999907_m1) (Applied Biosystems, Foster City, CA, USA) was used for template normalization. RT-PCR was performed in three independent experiments, duplicate determinations were carried out for each sample.

### Exosome/microvesicles (EMVs) isolation from conditioned medium of hPDLSCs and RR-MS-hPDLSCs

The basis for this method lays on the precipitation of EMVs using a commercial agglutinating agent exoquick TC (System Biosciences). The experiments were performed following the manufacturer’s instructions, in summary 2 ml of ExoQuick TC were added to 10 ml of conditioned medium recovered from hPDLSCs and RR-MS-hPDLSCs at 2^nd^ passage. The mix was incubated overnight at 4 °C without rotation, one centrifugation step was performed at 1,500 × g for 30 min to sediment the EMVs and the pellets were resuspended in 200 μl of PBS. The detection of EMVs whole homogenate proteins was used as a confirmation of the presence of release of EMVs in hPDLSCs and RR-MS-hPDLSCs.

### EMVs preparation for cytometric evaluation and fluorescence microscopy

EMVs were treated as reported in immunophenotypingof hPDLSCs and RR-MS-hPDLSCs section, briefly were incubated with anti CD90, anti-CD29 and anti-CD63.

For morphological observation, EMVs were cytospinned and after incubated with Alexa Fluor® 488 conjugate Wheat germ agglutinin (WGA) for 10 minutes at 37 °C. When labeling was completed, EMVs were washed in PBS, fixed with 4% paraformaldehyde and observed to fluorescence microscopy. The images were captured with optical fluorescence microscope (LEICA-8000B, Leica Microsystem, Milan, Italy).

### EMVs protein extraction for western blot analysis

Exosomes were resuspended in RIPA cold hypotonic lysis buffer (1x PBS, 1% Igepal, 0.5% sodium deoxycholate, 0.1% sodium dodecyl sulphate (SDS), 10 μg/ml phenylmethylsulfonyl fluoride (PMSF) and 10 μl/ml of Protease Inhibitor Cocktail (Sigma-Aldrich, Milan, Italy)). The level of recovered protein was measured spectrometrically according to the instructions of the manufacturer using the Bio-Rad (Hercules, CA, USA) Protein Assay (detergent compatible)^61^.

Proteins were separated on sodium dodecyl sulfate-polyacrylamide minigels and transferred onto PVDF membranes (Immobilon-P Transfer membrane, Millipore), blocked with PBS containing 5% nonfat dried milk (PM) for 45 min at room temperature, and subsequently probed at 4 °C overnight with specific antibodies, TGFβ (1:500 Abcam) and IL-10 (1:500; Santa Cruz Biotechnology, Inc) in 1x PBS, 5% (w/v) non fat dried milk, 0.1% Tween-20 (PMT). HRP-conjugated goat anti-rabbit IgG, HRP-conjugated goat anti-rat IgG were incubated as secondary antibody (1:2000; Santa Cruz Biotechnology Inc) for 1 h at room temperature. The relative expression of protein bands, was visualized using an enhanced chemiluminescence system (Luminata Western HRP Substrates, Millipore) and protein bands were acquired and quantified with ChemiDoc MP System (Bio-Rad) and a computer program UVIband-1D gel analysis software (Uvitec, Cambridge, UK) respectively. The protein bands were normalized to β-actin for quantification. Blots are representative of three separate and reproducible experiments. The statistical analysis was carried out on three repeated blots performed on separate experiments.

### Animals

Male C57BL/6 mice (Harlan Milan, Italy) 12 weeks of age and weighing 20–25 g were housed in individually ventilated cages with food and water *ad libitum*. The room was maintained at a constant temperature and humidity on a 12 h/12 h light/dark cycle.

### Ethics statement

All the animal experiments were carried out in strict accordance with the European Organization Guidelines for Animal Welfare. The protocol was approved by the Ministry of Health “General Direction of animal health and veterinary drug” (Authorization 621/2015- D.lgs 26/2014). Also, minimized number of animals were used for this experiment.

### Induction of Experimental Autoimmune Encephalomyelitis (EAE)

For this study, we have chosen male C57BL/6 as several studies have demonstrated that gender does not influence the incidence and disease course of EAE^62^,^63^. Specifically, according to Papenfuss TL. *et al*.^62^ sexual dimorphism in EAE incidence and clinical course exists in the SJL/J, B10.PL, ASW, PL/J and NZW/LAC strains and not in the C57BL/6 strain.

After anesthesia, induced with an anesthetic cocktail composed of tiletamine plus xylazine (10 ml/kg, ip), EAE was actively induced using Myelin Oligodendrocyte Glycoprotein peptide (MOG)35–55 (MEVGWYRSPFSRVVHLYRNGK; % peak area by HPLC ≥95, AnaSpec, EGT Corporate Headquarters, Fremont, CA, USA), according to Paschalidis *et al*.^64^. Mice were immunized subcutaneously with 300 μl/flank of the emulsion consisting of 300 μg of (MOG)_35-55_ in phosphate-buffered saline (PBS) mixed with an equal volume of Complete Freund’s Adjuvant (CFA) containing 300 μg heat-killed M. Tubercolosis H37Ra (Difco Laboratories Sparks, MD,USA). Immediately after (MOG)_35-55_ injection, the animals received an ip injection of 100 μl of B. Pertussis toxin (Sigma-Aldrich, Milan, Italy) (500 ng/100 μl, i.p), repeated 48 h later. The disease follows a course of progressive degeneration, with visible signs of pathology consisting of flaccidity of the tail and loss of motion of the hind legs.

### Experimental design

Mice were randomly allocated into the following groups (N = 55 total animals):

1. Naive group (N = 5): mice did not receive (MOG)_35-55_ or other treatment;
2. EAE group (N = 10): mice subjected to EAE that did not receive pharmacological treatment;
3. EAE + hPDLSCs-CM group obtained from RR-MS patients (N = 5): at the onset of disease signs, that normally occurs approximately 14 days after immunization, EAE mice were subjected to intravenous injection (i.v) into the tail vein with ≈1,600 μGof hPDLSCs-CM/mouse;
4. EAE + hPDLSCs-CM group obtained from healthy donors (N = 5): at the onset of disease signs, that normally occurs approximately 14 days after immunization, EAE mice were subjected to i.v into the tail vein with ≈1,300 μG of hPDLSCs-CM/mouse;
5. EAE + hPDLSCs-EMVs obtained from RR-MS patients group (N = 5): at the onset of disease signs, that normally occurs approximately 14 days after immunization, EAE mice were subjected to i.v into the tail vein with ≈24 μG of hPDLSCs-EMVs/mouse;
6. EAE + hPDLSCs-EMVs obtained from healthy donors group (N = 5): at the onset of disease signs, that normally occurs approximately 14 days after immunization, EAE mice were subjected to i.v into the tail vein with ≈24 μG of hPDLSCs-EMVs/mouse;
7. NAIVE + hPDLSCs-CM obtained from RR-MS patients group (N = 5): mice did not receive (MOG)_35-55_ but subjected to i.v into the tail vein with ≈1,600 μG of hPDLSCs-CM/mouse;
8. NAIVE + hPDLSCs-CM obtained from healthy donorsgroup (N = 5): mice did not receive (MOG)_35-55_ but subjected to i.v into the tail vein with ≈1,300 μG of hPDLSCs-CM/mouse;
9. NAIVE + hPDLSCs-EMVs obtained from RR-MS patients group (N = 5): mice did not receive (MOG)_35-55_ but subjected to i.v into the tail vein with ≈24 μG of hPDLSCs-EMVs/mouse;
10. NAIVE + hPDLSCs-EMVs obtained from healthy donors group (N = 5): mice did not receive (MOG)_35-55_ but subjected to i.v into the tail vein with ≈24 μG of hPDLSCs-EMVs/mouse.

At the end of the experiment, which occurred at the 28^th^ day from EAE-induction, all animals treated with were euthanized with i.p. of Tanax (5 ml/kg body weight). Spinal cord and spleen tissues were sampled and processed in order to evaluate parameters of disease.

### Clinical disease score and body weight evaluation

14 days after EAE induction, mice show the first signs of MS disease, characterized by loss of tail tonus, hind limb paralysis and body weight loss. Mice were daily weighed and observed for signs of EAE. Disease severity was evaluated with a 0–10 scoring system, according to Campbell AM *et al*.^65^ with 0 representing no disease and 10 representing death due to EAE. We have chosen this score system since it allows the assessment of more disease parameters. In addition, a scale that considered more discrete intervals (for example, scores of 0 to 10) typically has more capacity to detect statistical differences and is a more sensitive measure for mild disease activity than a scale with fewer intervals^66^.

In detail, observations of tail tonicity, gait, righting reflex and limb function were scored and summed to provide a daily disease score. Tail tone was scored as normal (0), distal loss of tone (+0.5), or completely loss of tone (+1). Gait was scored as normal (0), slightly abnormal (+1), moderately abnormal (+2) or severe (+3). Righting reflex tests were performed by placing the animal on its back and scoring the ability to return quickly to all four limbs. Righting reflex was scored as normal (0), slow (+0.5) or absent (+1). Function of each limb was scored independently as follows: normal (0), weak (+0.5), near paralysis (+1), or paralyzed (+1.5). To assess limb weakness. Mice were placed in an inverted position on a grid for 20 sec. Limb weakness was identified if animals repeatedly released the grid, or had the inability to grasp the grid. A limb was determined to be near paralysis if there was limited limb movement plus inability to hold the limb under the body and support weight during ambulation. A limb was considered paralyzed if was completely incapable of movement. Animal displaying both hindlimb and forelimb paralysis was scored as 9, and death due to EAE was scored as 10. The first measurement of clinical disease score and body weight were taken on the day of EAE- induction (day zero), and all the subsequent measurements were recorded every 24 hours until sacrifice. Also, the daily variation of these two parameters of disease has been expressed compared to day of EAE induction (day zero). The value day has been expressed as mean ± SEM of all animals for each experimental group.

### Light microscopy

At 28^th^ day of EAE-induction, spinal cord and spleen tissues were sampled and fixed in10% (w/v) in PBS-buffered formaldehyde, embedded in paraffin and then cut into sections 7 microns thick. The sections were deparaffinized with xylene, rehydrated, and stained with H&E to be studied by optical microscope (Leica microscope ICC50HD).

### Cytokine measurements in spinal cord

ELISA kit testing for IL-17 (MyBioSource ELISA kit, catalog no. MBS013248), TNF-α (eBioscience ELISA Ready-SET-Go!, catalog no. 88-7324-22), IFN-γ (Abcam ELISA kit, catalog no. ab100690), IL-6 (Abcam ELISA kit, catalog no. ab100712), IL-1β (MyBioSource ELISA kit, catalog no. MBS355354), and IL-10 (Abcam ELISA kit catalog, no. ab108870) were purchased to measure cytokine levels in spinal cord samples. The kits were used according to the manufacturer’s protocol. Experiment was repeated for three times.

### Immunohistochemical evaluation

After deparaffinization with xylene, sections of spinal cord and spleen samples were hydrated. Detection of CD4, Bax, IL-17, IFN-γ and IL-10 was carried out after boiling in citrate buffer 0.01 M pH 6 for 4 min. Endogenous peroxidase was quenched with 0.3% (v/v) hydrogen peroxide in 60% (v/v) methanol for 30 min. Nonspecific adsorption was minimized by incubating the section in 2% (v/v) normal goat serum in PBS for 20 min.

Sections were incubated overnight with:

- anti-CD4 polyclonal antibody (1:100 in PBS v/v; Santa Cruz Biotechnology, Inc);
- anti-Bax polyclonal antibody (1:100 in PBS v/v; Santa Cruz Biotechnology, Inc);
- anti-IL-17 polyclonal antibody (1:100 in PBS v/v; Abcam);
- anti-IFN-γ monoclonal antibody (1:100 in PBS v/v; Santa Cruz Biotechnology, Inc);
- anti-IL-10 polyclonal antibody (1:100 in PBS v/v; Santa Cruz Biotechnology, Inc);

Endogenous biotin or avidin binding sites were blocked by sequential incubation for 15 min with biotin and avidin (DBA, Milan, Italy), respectively. Sections were washed with PBS and incubated with secondary antibody. Specific labelling was detected with a biotin-conjugated goat anti-rabbit IgG and avidin–biotin peroxidase complex (Vectastain ABC kit, VECTOR). The immunostaining was developed with peroxidase substrate kit DAB (Vector Laboratories, Inc.) (brown color) and counterstaining with hematoxylin (blue background). To verify the binding specificity, some sections were also incubated with only the primary antibody (no secondary) or with only the secondary antibody (no primary). In these cases no positive staining was found in the sections, indicating that the immunoreaction was positive in all the experiments carried out. All sections were obtained using light microscopy (LEICA DM 2000 combined with LEICA ICC50 HD camera). Leica Application Suite V4.2.0 software was used as image computer program to acquire immunohistochemical pictures.

### Western blot analysis

All the extraction procedures were performed on ice using ice-cold reagents. In brief, spinal cord and spleen tissues were suspended in extraction buffer containing 0.32 M sucrose, 10 mM Tris-HCl, pH 7.4, 1 mM EGTA, 2 mM EDTA, 5 mM NaN_3_, 10 mM 2-mercaptoethanol, 50 mM NaF, protease inhibitor tablets (Roche Applied Science, Monza, Italy), and they were homogenized at the highest setting for 2 min. The homogenates were chilled on ice for 15 min and then centrifuged at 1,000 g for 10 min at 4 °C, and the supernatant was collected to evaluate content of cytoplasmic proteins. The pellets were suspended in the supplied complete lysis buffer containing 1% Triton X-100, 150 mM NaCl, 10 mM Tris-HCl, pH 7.4, 1 mM EGTA, 1 mM EDTA protease inhibitors (Roche), and then were centrifuged for 30 min at 15,000 g at 4 °C. Then, supernatant containing nuclear extract was collected to evaluate the content of nuclear proteins. Supernatants were stored at −80 °C until use. Protein concentration in homogenate was estimated by Bio-Rad Protein Assay (Bio-Rad, Segrate, Italy) using BSA as standard, and 20 μg of cytosol and nuclear extract from each sample were analyzed. Proteins were separated on sodium dodecyl sulfate-polyacrylamide minigels and transferred onto PVDF membranes (Immobilon-P Transfer membrane, Millipore), blocked with PBS containing 5% nonfat dried milk (PM) for 45 min at room temperature, and subsequently probed at 4 °C overnight with specific antibodies, cleaved-caspase 3 (1:500; Cell Signaling Technology), CD4 (1:500 Abcam), STAT-1 (1:500 Abcam) and p53 (1:2000; Abcam) in 1x PBS, 5% (w/v) non fat dried milk, 0.1% Tween-20 (PMT). HRP-conjugated goat anti-mouse IgG, HRP-conjugated goat anti-rabbit IgG or HRP-conjugated chicken anti-rat were incubated as secondary antibody (1:2000; Santa Cruz Biotechnology Inc) for 1 h at room temperature. To ascertain that blots were loaded with equal amounts of protein lysates, they were also incubated with antibody for GAPDH HRP Conjugated (1:1000; Cell Signaling Technology). The relative expression of protein bands, was visualized using an enhanced chemiluminescence system (Luminata Western HRP Substrates, Millipore) and protein bands were acquired and quantified with ChemiDoc MP System (Bio-Rad) and a computer program (ImageJ software) respectively. Blots are representative of three separate and reproducible experiments. The statistical analysis was carried out on three repeated blots performed on separate experiments.

### Statistical analysis

GraphPad Prism version 6.0 program (GraphPad Software, La Jolla, CA) was used for statistical analysis of the data. The results were analyzed by unpaired Student’s *t*-test. A *p* value of <0.05 was considered to be statistically significant. All results achieved are presented as the means ± S.E.M of n experiments. The factors under investigation were the time elapsed and the mRNA expression for MTT assay and the differentiation ability respectively. Data were expressed as means and standard deviation of the recorded dependent variables: the optical density (MTT assay) and mRNA expression (osteogenic and adipogenic differentiation). The differences among the levels of the two factors under investigation were evaluated performing four distinct two-way-ANOVA tests, one for each experiment. Tukey tests were applied for pairwise comparisons. The results for clinical disease score, body weight and western blot analysis were statistically analyzed using one-way analysis of variance followed by a Bonferroni post hoc test for multiple comparisons. A *p* value less than or equal to 0.05 was considered significant. Results are expressed as the mean ± S.D of n experiments.

## Additional Information

**How to cite this article**: Rajan, T. S. *et al*. The secretome of periodontal ligament stem cells from MS patients protects against EAE. *Sci. Rep.* **6**, 38743; doi: 10.1038/srep38743 (2016).

**Publisher's note:** Springer Nature remains neutral with regard to jurisdictional claims in published maps and institutional affiliations.

## Supplementary Material

Supplementary Information
