# Supplementary material for: The secretome of periodontal ligament stem cells from MS patients protects against EAE
Source: Sci Rep. 2016 Dec 7;6:38743. doi: 10.1038/srep38743 (PMC5141419; doi:10.1038/srep38743)
Supplement: Supplementary Information [file srep38743-s1.pdf]

The secretome of periodontal ligament stem cells from MS patients protects against EAE

Thangavelu Soundara Rajan[1*], Sabrina Giacoppo[1*], Francesca Diomede[2], Patrizia Ballerini[3], Michele Paolantonio[2], Marco Marchisio[4], Adriano Piattelli[2], Placido Bramanti[1], Emanuela Mazzon[1], Oriana Trubiani[2]

[1] IRCCS Centro Neurolesi "Bonino-Pulejo", Via Provinciale Palermo, contrada Casazza, 98124, Messina, Italy.
[2] Stem Cells and Regenerative Medicine Laboratory, Department of Medical, Oral and Biotechnological Sciences, University "G. d'Annunzio", Chieti-Pescara, via dei Vestini, 31, 66100, Chieti, Italy.
[3] Department of Psychological, Health and Territorial Sciences, University "G. d'Annunzio" Chieti-Pescara, via dei Vestini, 31, 66100, Chieti, Italy.
[4] Department of Medicine and Aging Science, University "G. d'Annunzio" Chieti-Pescara, via dei Vestini, 31, 66100, Chieti, Italy.

*These authors contributed equally to this work

Correspondence and requests for materials should be addressed to:
Dr.Emanuela Mazzon PhD
Email: emazzon.irccs@gmail.com

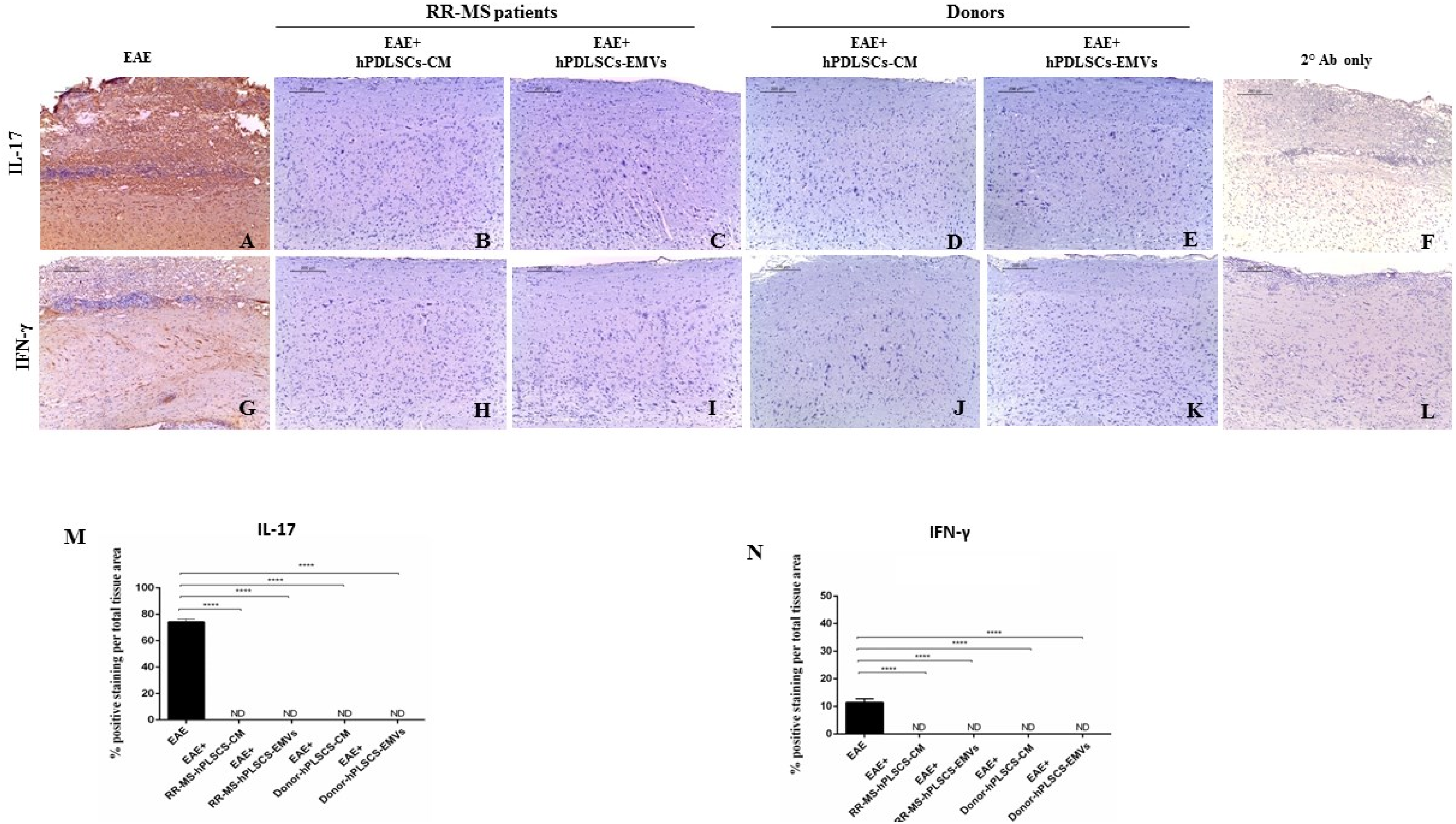

Supplementary Figure 1. Immunohistochemical evaluation for pro-inflammatory IL-17 and IFN-γ in the spinal cord. IL-17 [**A**; densitometric analysis **M**] and IFN-γ [**G**; densitometric analysis **N**] expression revealed marked positive staining of these markers in EAE mice, while EAE mice administered with hPDLSCs-CM or hPDLSCs-EMVs derived from MS patients [**B**, **C** and **H**, **I**, respectively] and donors [**D**, **E** and **J**, **K**, respectively] showed negative staining. ****$p<0.0001$. **F** and **L** only secondary antibody. Magnification 10X. ND-not detectable.

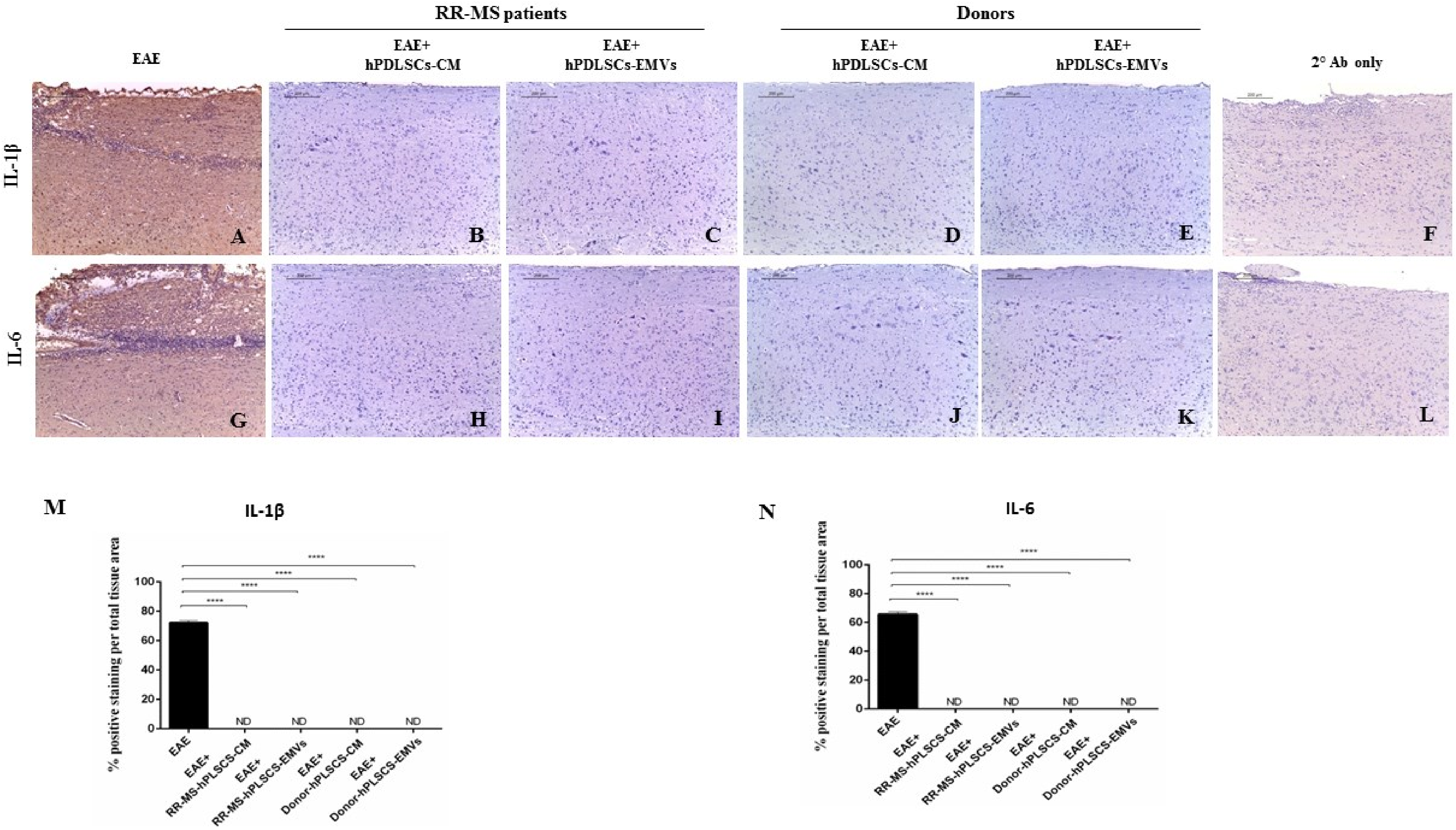

Supplementary Figure 2. Immunohistochemical evaluation for pro-inflammatory IL-1β and IL-6 in the spinal cord. IL-1β [**A**; densitometric analysis **M**] and IL-6 [**G**; densitometric analysis **N**] expression revealed marked positive staining of these markers in EAE mice, while EAE mice administered with hPDLSCs-CM or hPDLSCs-EMVs derived from MS patients [**B**, **C** and **H**, **I**, respectively] and donors [**D**, **E** and **J**, **K**, respectively] showed negative staining. ****$p<0.0001$. **F** and **L** only secondary antibody. Magnification 10X. ND-not detectable.

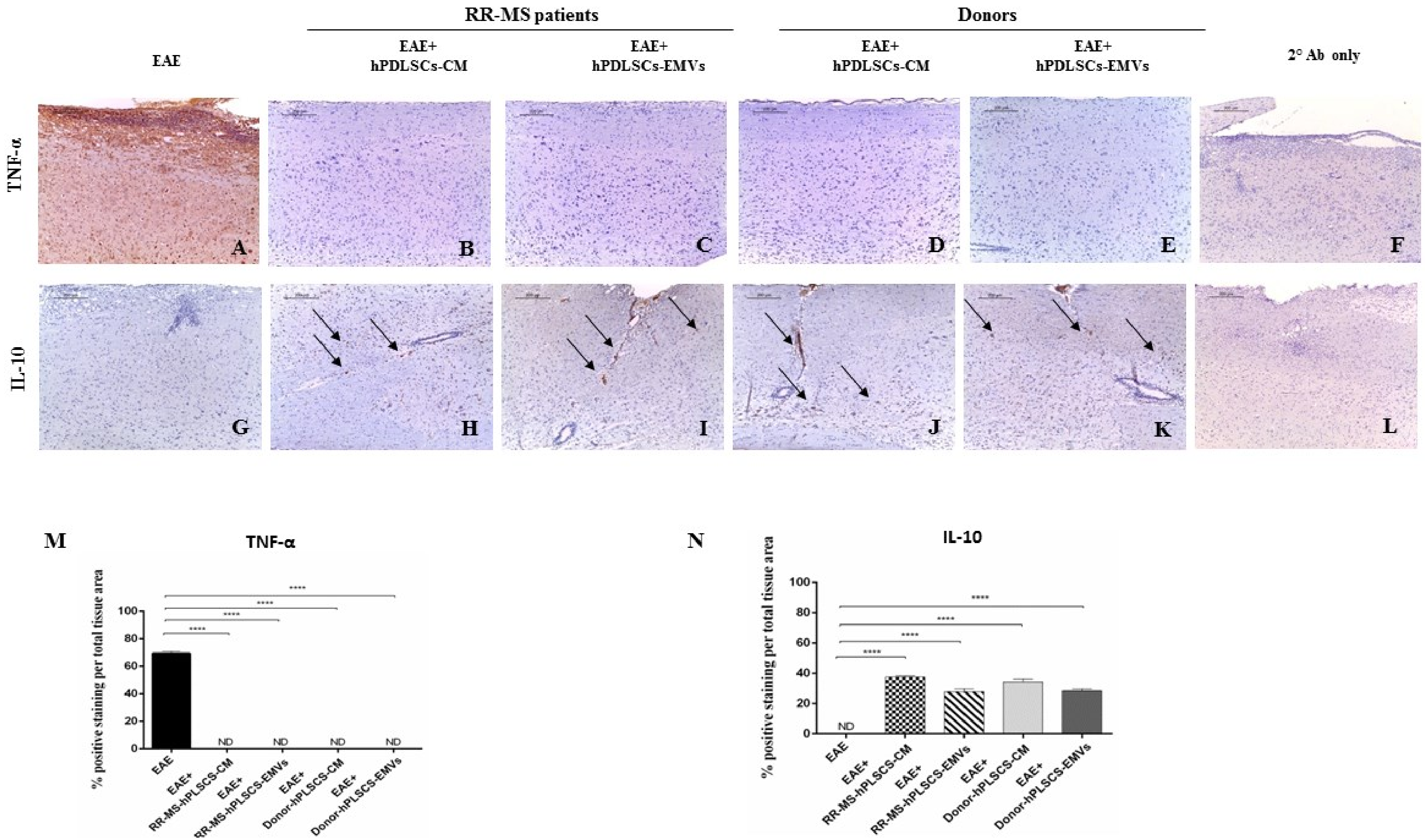

Supplementary Figure 3. Immunohistochemical evaluation for pro-inflammatory TNF-α and anti-inflammatory IL-10 in the spinal cord. Pro-inflammatory TNF- α [**A**; densitometric analysis **M**] revealed higher tissue level expression in EAE mice, while significant reduction was noticed in EAE mice administered with hPDLSCs-CM or hPDLSCs-EMVs derived from RR-MS patients [**B** and **C**, respectively] and donors [**D** and **E**, respectively]. EAE sections did not stain for anti-inflammatory IL-10 antibody [**G;** densitometric analysis **N**], while significant positive IL-10 staining was noticed (indicated by arrows) in the EAE group treated with hPDLSCs-CM or hPDLSCs-EMVs obtained from MS patients [**H** and **I**, respectively] and donors [**J** and **K**, respectively]. ****p<0.0001. **F** and **L** only secondary antibody. Magnification 10X. ND-not detectable

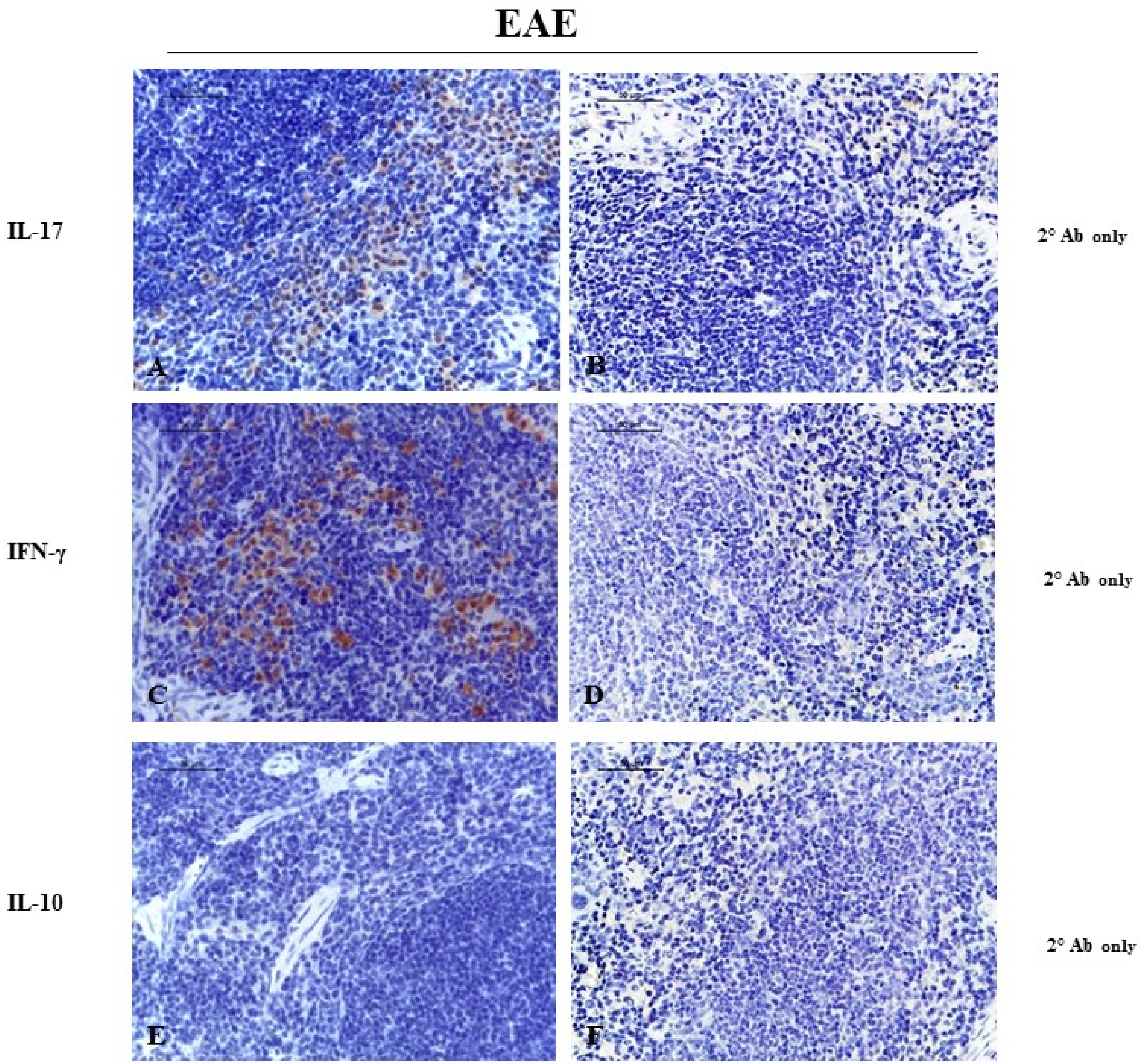

Supplementary Figure 4. Immunohistochemical evaluation for inflammatory cytokines IL-17, IFN-γ and IL-10 with respective secondary antibody control in the spleen. Representative images showed positive staining for IL-17 (A), IFN-γ (C), and IL-10 (E) in the spleen. Negative staining was observed in tissue sections stained with only secondary antibody for IL-17 (B), IFN-γ (D) and IL-10 (F). Magnification 40X.
